# Macular Choroidal Thickness and the Risk of Referable Diabetic Retinopathy in Type 2 Diabetes: A 2-Year Longitudinal Study

**DOI:** 10.1167/iovs.63.4.9

**Published:** 2022-04-14

**Authors:** Wei Wang, Longyue Li, Jun Wang, Yifan Chen, Xiong Kun, Xia Gong, Daheng Wei, Dongning Wang, Xiaolin Liang, Hua Liu, Wenyong Huang

**Affiliations:** 1 Zhongshan Ophthalmic Center, State Key Laboratory of Ophthalmology, Sun Yat-Sen University, Guangzhou, People's Republic of China; 2 School of Medicine, Sun Yat-Sen University, Guangzhou, People's Republic of China; 3 John Radcliffe Hospital, Oxford University Hospitals NHS Foundation Trust, Oxford, United Kingdom; 4 Institute of Eyes, Jinzhou Medical University, Jinzhou, Liaoning, People's Republic of China

**Keywords:** referable diabetic retinopathy, choroidal thickness, swept source optical coherence tomography, prediction, Chinese

## Abstract

**Purpose:**

The purpose of this study was to evaluate the associations between choroidal thickness (CT) and the 2-year incidence of referable diabetic retinopathy (RDR).

**Methods:**

This was a prospective cohort study. Patients with type 2 diabetes in Guangzhou, China, aged 30 to 80 years underwent comprehensive examinations, including standard 7-field fundus photography. Macular CT was measured using a commercial swept-source optical coherence tomography (SS-OCT) device (DRI OCT Triton; Topcon, Tokyo, Japan). The relative risk (RR) with 95% confidence intervals (CIs) was used to quantify the association between CT and new-onset RDR. The prognostic value of CT was assessed using the area under the receiver operating characteristic curve (AUC), net reclassification improvement (NRI), and integrated discrimination improvement (IDI).

**Results:**

A total of 1345 patients with diabetes were included in the study, and 120 (8.92%) of them had newly developed RDR at the 2-year follow-up. After adjusting for other factors, the increased RDR risk was associated with greater HbA1c (RR = 1.35, 95% CI = 1.17–1.55, *P* < 0.001), higher systolic blood pressure (SBP; RR = 1.02, 95% CI = 1.01–1.03, *P* = 0.005), lower triglyceride (TG) level (RR = 0.81, 95% CI = 0.69–0.96, *P* = 0.015), presence of diabetic retinopathy (DR; RR = 8.16, 95% CI = 4.47–14.89, *P* < 0.001), and thinner average CT (RR = 0.903, 95% CI = 0.871–0.935, *P* < 0.001). The addition of average CT improved NRI (0.464 ± 0.096, *P* < 0.001) and IDI (0.0321 ± 0.0068, *P* < 0.001) for risk of RDR, and it also improved the AUC from 0.708 (95% CI = 0.659–0.757) to 0.761 (95% CI = 0.719–0.804).

**Conclusions:**

CT thinning measured by SS-OCT is an early imaging biomarker for the development of RDR, suggesting that alterations in CT play an essential role in DR occurrence.

Globally, 9.3% of adults aged 20 to 79 years have diabetes mellitus (DM), and 35% of them were suffering from diabetic retinopathy (DR).^1^ Given DR's lack of obvious symptoms and irreversibility, early identification and appropriate treatment are crucial for preventing blindness from DR.^2^ The choroid is a highly vascularized tissue, which receives 95% of the blood in the eyeballs, and histological studies have shown that hyperglycemia leads to choroidal capillary obstruction, tortuosity, and nonperfused areas, as well as retinopathy.^3^ In patients with diabetes, choroidal abnormalities had been confirmed through indocyanine green angiography and ultrasonography.^4^ Several studies have indicated that choroidal impairment plays an essential role in the development of DR, so it is crucial to evaluate the value of choroidal measurements in predicting DR risk.

Optical coherence tomography (OCT) has been adopted as an effective tool for imaging choroid in vivo,^5^ and OCT-derived choroidal thickness (CT) has been proposed as a quantitative indicator of choroid structure and function. Several studies in the last decade have analyzed CT changes in patients with diabetes, but the results were varied, reporting CT thinning, no change, and even thickening.^6^^–^^10^ Notably, some found that CT was thinner in patients with DM without DR than in normal controls, suggesting that choroidal abnormalities may occur before retinopathy.^11^ Because these studies were all cross-sectional and included small sample sizes, making it difficult to infer causation, a longitudinal study is urgently needed to clarify whether CT is an early marker for predicting the risk of DR.

The latest swept-source OCT (SS-OCT) enables accurate CT measurements by detecting more choroidal detail and a clearer choroid-sclera interface than traditional OCT techniques.^12^ Using the SS-OCT technique equipped with an automatic algorithm, this prospective, longitudinal study quantified CT in a large sample of patients with type 2 DM and analyzed its relationship with the subsequent risk of referable DR (RDR) during a 2-year follow-up period.

## Materials and Methods

### Study Population

The Guangzhou Diabetic Eye Study (GDES) is an ongoing, community-based, prospective cohort study (ISRCTN registry no: 15853192) that recruited patients with type 2 DM from community health centers registered in the Yuexiu district in Guangzhou.^13^ All examinations were performed at the Zhongshan Ophthalmic Center, affiliated with Sun Yat-sen University. The project followed the principles of the Declaration of Helsinki and was approved by the Ethics Committee of Zhongshan Ophthalmic Center (2017KYPJ094). All participants signed a written informed consent form before enrolling in the study.

The inclusion criteria for participants in this study were as follows: type 2 DM, from 30 to 80 years old, no history of ocular treatment (naïve to treatment), and best corrected visual acuity (BCVA) of 20/40 or better so that participants could complete the eye examinations. Participants were excluded if any of the following conditions were present: high refractive error, including spherical equivalent of –6 diopters (D) or less, astigmatism of 1.5 D or greater, axial length (AL) of 26.0 mm or longer; history of serious systemic disease other than diabetes, such as uncontrolled hypertension, severe cardiovascular disease, malignancy, or nephritis; history of systemic surgery or renal dialysis; or current glaucoma, vitreoretinal disease, or amblyopia. Patients were also excluded if they had a history of retinal laser or intraocular injections; glaucoma, cataract, or corneal refractive surgery; or poor fundus or OCT image quality because of refractive media abnormalities (e.g. moderate to severe cataracts, corneal ulcers or severe pterygium, low OCT image signal strength, and poor gaze).

### Basic Information and Laboratory Tests

A standardized questionnaire was used to collect information about age, sex, duration of diabetes, history of systemic diseases, history of medication use, and lifestyle. Experienced investigators measured height, weight, systolic blood pressure (SBP) and diastolic blood pressure (DBP). Trained nurses collected urine and blood samples from all study subjects, and the following parameters were obtained by standard testing procedures: glycosylated hemoglobin (HbA1c), serum creatinine, total cholesterol (TC), HDL cholesterol (HDL-C), LDL cholesterol (LDL-C), triglycerides (TGs), C-reactive protein (CRP), and microalbuminuria (MAU).

### Basic Eye Examination

All study subjects underwent comprehensive ocular examinations. An optometrist measured BCVA using an Early Treatment of Diabetic Retinopathy Study (ETDRS) LogMAR E visual acuity meter (Precision Vision, Villa Park, IL, USA). Ocular biometric parameters, including AL, central corneal thickness, central anterior chamber depth (ACD), and lens thickness, were measured by an experienced technician using a Lenstar LS900 biometer (HAAG-Streit AG, Koeniz, Switzerland). Refraction was measured after pupil dilation by an optometrist using an automated optometry device (Topcon KR8800; Topcon Corporation, Tokyo, Japan).

### Fundus Photography and Definitions of Outcomes

Seven standard fundus photographs were taken for each eye after pupil dilation, according to ETDRS protocol, using a digital fundus camera (Canon CX-1; Canon Inc., Tokyo, Japan). The fundus images were graded according to the modified Airlie House classification. Retinopathy severity was classified as no DR (NDR), mild nonproliferative diabetic retinopathy (NPDR), moderate NPDR, severe NPDR, or proliferative diabetic retinopathy (PDR). Diabetic macular edema (DME) was confirmed with OCT images. RDR was defined as the presence of moderate NPDR, severe NPDR, PDR, or DME.

The primary outcome was new-onset RDR at 2 years, defined as patients with NDR, or mild NPDR, and without DME at baseline who developed RDR over the 2-year follow-up. This end point is clinically useful because individuals in DR screening programs with RDR are referred to an ophthalmologist for evaluation, whereas individuals with NDR or mild NPDR continue to be screened in primary care. The secondary outcome was new-onset DME during the 2-year follow-up.

### SS-OCT Examination and CT Measurement

**Figure 1.**
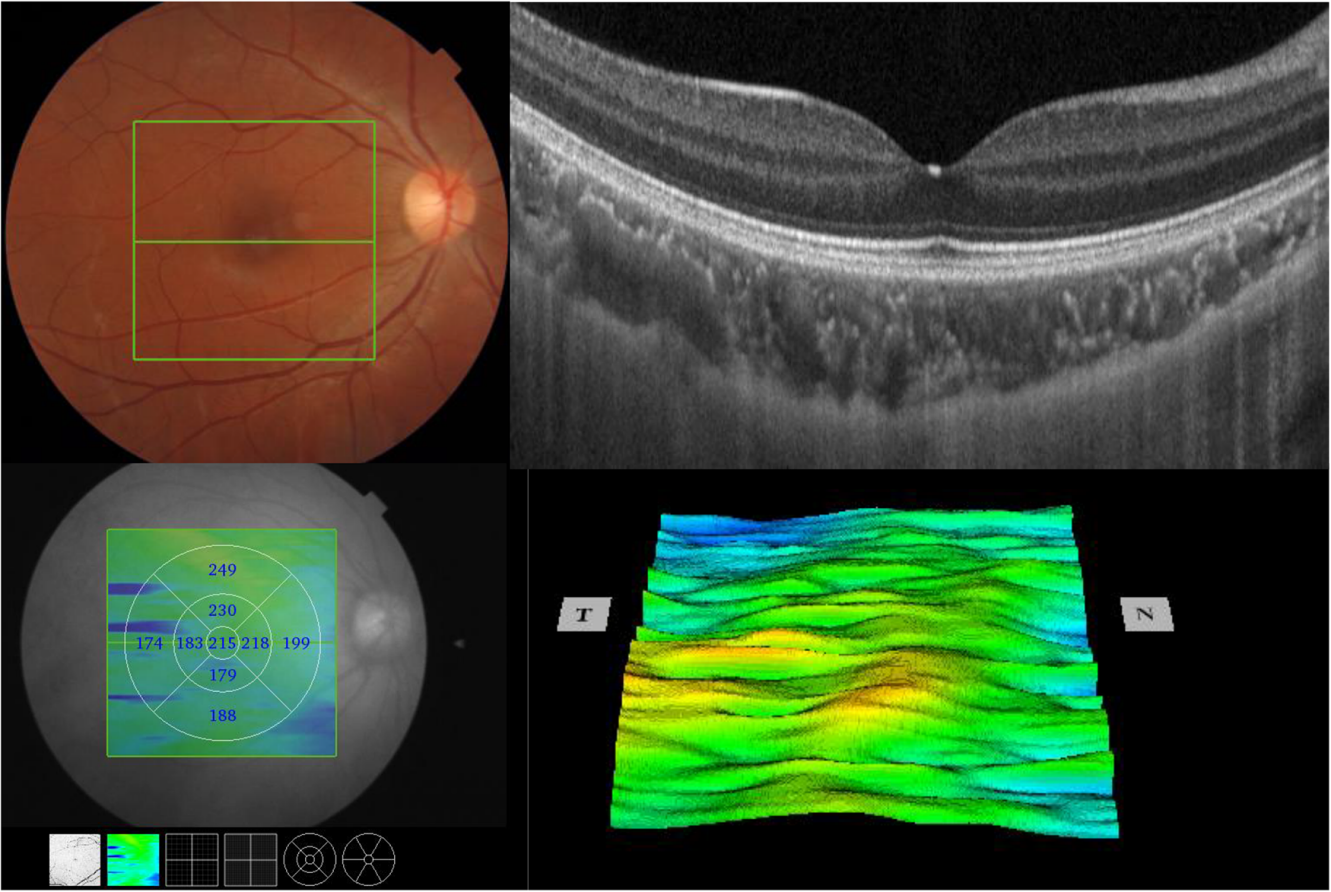
Quantification of choroidal thickness (CT) in macular regions by swept-source optical coherence tomography. Each region's CT was presented using the ETDRS nine-pattern grid, including the central, inner superior, inner nasal, inner inferior, inner temporal, outer superior, outer nasal, outer inferior, and outer temporal fields.

Choroidal imaging was performed using a commercially available SS-OCT device (DRI OCT Triton; Topcon, Tokyo, Japan). This instrument has a scanning speed of 100,000 A-scans/second and a central wavelength of 1050 nm light source, which is particularly suitable for imaging deep structures. The 3D Macular Cube 7 × 7 mm scan mode was used to image the retina during a 1.3-second scan time, centered on the macula, with a scan density of 512 A-scans × 512 B-scans. The built-in software automatically segmented each layer of the retinal and choroidal junction and measured each layer's thickness in an automated manner. Each region's CT was presented using the ETDRS nine-pattern grid, which divided the macula into inner and outer rings, including the central, inner superior, inner nasal, inner inferior, inner temporal, outer superior, outer nasal, outer inferior, and outer temporal fields (Fig. 1). In addition, the average CT from the nine grids was calculated automatically and displayed.

All OCT scans were performed by the same trained technologist, who was unaware of the patients’ diagnosis. The results of the automatic segmentation results were evaluated by image experts, and manual adjustments were made if segmentation errors existed. Participant data were blinded for image processing. Only high-quality scanned images were used for analysis, and images with the following conditions were excluded: image quality score <60, poor resolution, blink or residual motion artifacts, image decentering, poor contrast because of refractive media opacity, poor focus, or uncorrectable segmentation errors.

### Statistical Analysis

Outcomes were considered at the person level. Data from only one eye were included in the final analysis; if the DR grading was different in the two eyes, the more severe one was selected, and if the grading was the same in both eyes, the right eye was selected. Categorical variables were expressed as numbers (percentages), normally distributed continuous variables as mean ± standard deviation (SD), and non-normally distributed continuous variables as median (interquartile spacing).

First, the Kolmogorov–Smirnov test was used to confirm the normal distribution. A *t*-test or chi-square test was used to assess between-group differences in demographic, systemic, and ocular parameters for the analysis of RDR risk factors. A Bonferroni correction was performed for the CT measurements, and a *P* value < 0.005 (0.05/10) was considered statistically significant. Next, the relationships between the outcomes and the average CT at baseline were analyzed by univariable and stepwise multivariable logistic regression analysis. Predictors with *P* < 0.10 in univariable regressions were entered into the subsequent multivariable regression. Third, the relationship between the CT measurement in each ETDRS grid was assessed by univariable and multivariable logistic regression analyses. Fourth, the receiver operating characteristic (ROC) curve was constructed, and the area under the receiver operating characteristic curve (AUC; 95% confidence interval [CI]) was calculated to evaluate the performance of independent predictors for discriminating patients with high-risk RDR. Differences in the AUC before and after adding average CT to the predictive model were calculated and compared. Net reclassification improvement (NRI) and integrated discrimination improvement (IDI) were also estimated to evaluate the additive value of average CT in predicting RDR. The NRI and IDI were able to quantify the improvement in useful risk assessments when new predictors were combined into the conventional model. Finally, a systematic literature search was performed to identify previous studies on CT changes in diabetes based on SS-OCT and an automatic algorithm. The data were retrieved and pooled using random-effect models. A *P* value < 0.05 was considered statistically significant, except when otherwise specified. All data were processed and analyzed using Stata software (version 16.0; Stata Corp., College Station, TX, USA).

## Results

### Baseline Characteristics of the Study Population

**Figure 2.**
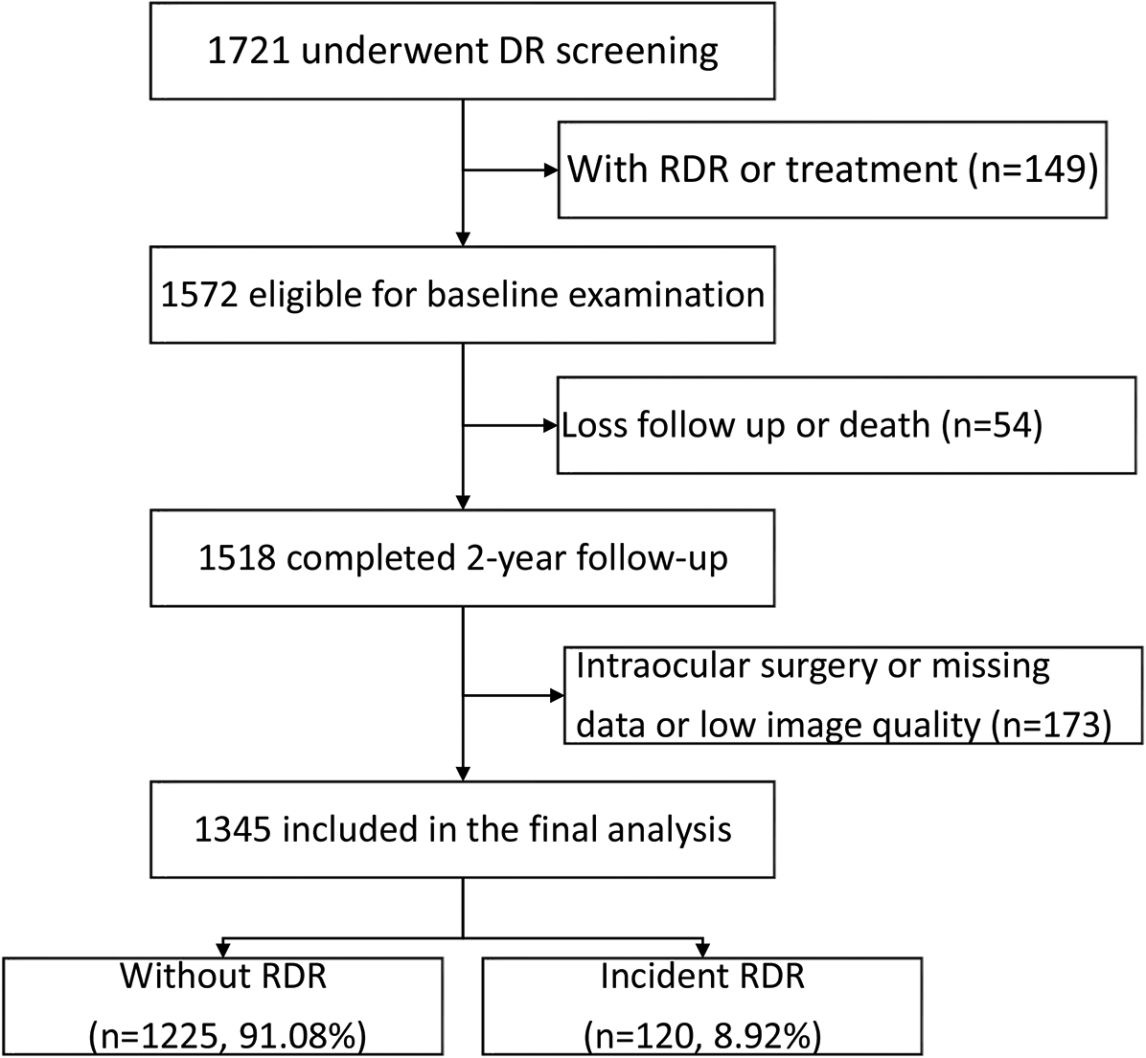
Flowchart of participants selection in the 2-year cohort study. RDR = referable diabetic retinopathy.

Figure 2 shows the process used to identify the study population. A total of 1721 patients with diabetes were recruited for screening, and patients with RDR (*n* = 149) and those lost to follow-up or death (*n* = 54) were excluded. A total of 173 patients were excluded because they had undergone intraocular surgery, had low-quality images, or were missing data during the 2-year follow-up period. A total of 1345 participants (1345 eyes) were included in the analysis (response rate = 88.6%), and 120 (8.92%) of them had newly developed RDR at the 2-year follow-up. Of the included participants, seven (0.52%) developed DME during the follow-up period. Compared to those who were excluded, the people included in the study were younger and had lower SBP and DBP, lower serum creatine, better BCVA, shorter AL and ACD, and thicker lenses (all *P* values < 0.05). The included and excluded subjects were similar regarding sex, HbA1c level, HDL-c, and DR status (Supplementary Table S1).

Table 1 lists the basic characteristics of the study population according to RDR status at the 2-year follow-up. Compared with stable subjects, those with new-onset RDR at the follow-up period were older and had diabetes longer. They also had higher Hba1C and SBP levels, worse BCVA, and more DR at baseline. The two groups were similar in terms of other baseline characteristics, such as sex, cholesterol, creatinine, HDL-C, LDL-C, and TG (see Table 1).

**Table 1.** Baseline Characteristics of the Included Subjects by Incident of Referable Diabetic Retinopathy Within 2 Years

		Incident RDR		
Variable	All	No	Yes	*P* Value
No. of subjects	1345	1225 (91.08%)	120 (8.92%)	–
Female, %	792 (58.88%)	718 (58.61%)	74 (61.67%)	0.516
Age, year	63.8 ± 7.4	63.7 ± 7.4	65.2 ± 7.1	0.025
DM duration, year	8.4 ± 6.5	8.3 ± 6.5	10.1 ± 6.7	0.004
HbA1c, %	6.74 ± 1.17	6.70 ± 1.14	7.19 ± 1.45	<0.001
SBP, mm Hg	133.6 ± 17.8	133.1 ± 17.8	139.0 ± 17.3	0.001
DBP, mm Hg	70.8 ± 9.9	70.9 ± 9.8	70.1 ± 10.4	0.434
Cholesterol, mmol/L	4.79 ± 1.06	4.80 ± 1.07	4.71 ± 0.96	0.374
Creatinine, µmol/L	69.4 ± 19.0	69.5 ± 18.6	68.8 ± 22.2	0.723
HDL-C, mmol/L	1.33 ± 0.41	1.33 ± 0.41	1.34 ± 0.37	0.755
LDL-C, mmol/L	3.05 ± 0.94	3.05 ± 0.94	3.07 ± 0.89	0.819
TG, mmol/L	2.29 ± 1.63	2.32 ± 1.68	2.00 ± 0.95	0.041
CRP, mg/L	2.45 ± 6.18	2.46 ± 6.38	2.34 ± 3.50	0.839
MAU, mg/mL	4.06 ± 15.73	3.82 ± 13.76	6.50 ± 28.88	0.076
BCVA, logMAR	0.19 ± 0.12	0.19 ± 0.11	0.25 ± 0.14	<0.001
IOP, mm Hg	16.3 ± 2.8	16.3 ± 2.8	16.3 ± 2.5	0.978
CCT, µm	546.8 ± 31.2	546.5 ± 31.5	549.4 ± 28.9	0.329
Axial length, mm	23.4 ± 0.9	23.4 ± 0.9	23.5 ± 0.9	0.639
ACD, mm	2.5 ± 0.5	2.5 ± 0.4	2.5 ± 0.6	0.609
Len thickness, mm	4.7 ± 0.3	4.7 ± 0.3	4.7 ± 0.4	0.197
Corneal diameter, mm	11.6 ± 0.4	11.6 ± 0.4	11.6 ± 0.4	0.572
Mild non-proliferative DR at baseline, %	65 (4.83%)	41 (3.35%)	24 (20%)	<0.001

DM = diabetes mellitus; SBP = systolic blood pressure; D = diopters; TG = triglyceride; HDL-C = high-density lipoprotein cholesterol; CCT = central corneal thickness; DR = diabetic retinopathy.

### Baseline CT Distribution

Table 2 reflects the distribution of the baseline CT at each ETDRS grid for the participants according to the RDR status at the end point. CT was significantly thinner in patients with new-onset RDR than in non-RDR patients, with an average CT of 166.3 ± 55.6 vs. 199.3 ± 62.5 (*P* < 0.001) and a CT in the central field of 183.1 ± 65.1 vs. 218.2 ± 72.3 (*P* < 0.001). Similar results were obtained for other regions (see Table 2). The participants who developed DME during the follow-up period had a thinner average CT than those without DME, but the difference did not reach a significant level (195.6 ± 61.3 vs. 196.4 ± 62.6, *P* = 0.972). Similarly, the CT in the central field was thinner in patients with new-onset DME than in non-DME patients (205.6 ± 65.4 vs. 215.2 ± 72.4, *P* = 0.727), although this result was not statistically significant (Supplementary Table S2).

**Table 2.** Baseline Distribution of Choroidal Thickness in Macular Region by Incident of Referable Diabetic Retinopathy at 2-Year Follow-Up

		Incident RDR		
Characteristics	All	No	Yes	*P* Value^*^
Outer superior, µm	204.3 ± 65.0	207.5 ± 64.6	172.4 ± 59.7	<0.0001
Inner superior, µm	214.4 ± 68.8	217.4 ± 68.7	183.8 ± 62.0	<0.0001
Outer temporal, µm	184.6 ± 58.7	187.4 ± 58.5	155.5 ± 52.2	<0.0001
Inner temporal, µm	206.9 ± 65.5	210.1 ± 65.4	175.0 ± 57.8	<0.0001
Central field, µm	215.1 ± 72.3	218.2 ± 72.3	183.1 ± 65.1	<0.0001
Inner nasal, µm	202.0 ± 74.2	205.0 ± 74.0	172.2 ± 69.6	<0.0001
Outer nasal, µm	160.0 ± 70.7	162.7 ± 70.6	132.8 ± 65.6	<0.0001
Inner inferior, µm	201.7 ± 74.4	204.6 ± 74.2	171.6 ± 70.0	<0.0001
Outer inferior, µm	178.9 ± 70.5	181.8 ± 70.4	149.8 ± 65.3	<0.0001
Average, µm	196.4 ± 62.6	199.3 ± 62.5	166.3 ± 55.6	<0.0001

RDR = referable diabetic retinopathy.

* *P* < 0.005 is considered as statistically significant by Bonferroni correction.

### Associations Between CT Measurements and New-Onset RDR

Univariable and stepwise multivariable logistic analyses were performed to explore the potential parameter at baseline for predicting the 2-year risk of RDR occurrence (Table 3). Age, duration of diabetes, HbA1c level, TG, MAU, DR status, and average CT were significant in the univariable model and were entered into the stepwise multivariable model. After adjusting for other factors, the increased RDR risk was associated with greater HbA1c (RR = 1.35, 95% CI = 1.17–1.55, *P* < 0.001), higher SBP (RR = 1.02, 95% CI = 1.01–1.03, *P* = 0.005), lower TG level (RR = 0.81, 95% CI = 0.69–0.96, *P* = 0.015), presence of DR (RR = 8.16, 95% CI = 4.47–14.89, *P* < 0.001), and thinner average CT (RR = 0.903, 95% CI = 0.871–0.935, *P* < 0.001).

**Table 3.** Univariable and Stepwise Multivariable Logistic Analyses of the Potential Predictors at Baseline for the 2-Year Risk of Incident RDR

	Univariable Model		Stepwise Multivariable Model	
Parameters at Baseline	RR (95% CI)	*P* Value	RR (95% CI)	*P* Value
Per 10-year increase in age	1.36 (1.04 to 1.78)	0.025		
Male versus female	0.88 (0.60 to 1.29)	0.517		
Per 1-year increase in diabetes duration	1.04 (1.01 to 1.07)	0.004		
Per % increase in HbA1c level	1.33 (1.16 to 1.51)	<0.001	1.35 (1.17 to 1.55)	<0.001
Per 1-mm Hg increase in systolic blood pressure	1.02 (1.01 to 1.03)	0.001	1.02 (1.01 to 1.03)	0.005
Per 1-mmHg increase in diastolic blood pressure	0.99 (0.97 to 1.01)	0.434		
Per 1-mmol/L increase in total cholesterol	0.92 (0.77 to 1.10)	0.373		
Per 1-mmol/L increase in serum creatinine	1.00 (0.99 to 1.01)	0.722		
Per 1-mmol/L increase in HDL-C level	1.07 (0.69 to 1.68)	0.755		
Per 1-mmol/L increase in LDL-C level	1.02 (0.84 to 1.25)	0.819		
Per 1-mmol/L increase in TG level	0.86 (0.74 to 0.99)	0.041	0.81 (0.69 to 0.96)	0.015
Per 1-mg/L increase in C-reactive protein	1.00 (0.96 to 1.03)	0.840		
Per 1-mg/mL increase in microalbuminuria	1.01 (1.00 to 1.02)	0.094		
Per 1-mm Hg increase in intraocular pressure	1.00 (0.93 to 1.07)	0.978		
Per 1-µm increase in central corneal thickness	1.00 (1.00 to 1.01)	0.329		
Per 1-mm increase in axial length	1.05 (0.85 to 1.30)	0.639		
Per 1-mm increase in anterior chamber depth	1.11 (0.76 to 1.62)	0.609		
Per 1-mm increase in lens thickness	1.48 (0.82 to 2.69)	0.197		
Per 1-mm increase in corneal diameter	1.15 (0.71 to 1.84)	0.571		
DR status at baseline (present versus without)	7.22 (4.19 to 12.45)	<0.001	8.16 (4.47 to 14.89)	<0.001
Per 10-µm increase in average choroidal thickness	0.910 (0.880 to 0.942)	<0.001	0.903 (0.871 to 0.935)	<0.001

RDR = referable diabetic retinopathy.

After correcting for the other factors, a significant association was seen between the CT in the central field and RDR risk (RR = 0.922, 95% CI = 0.895–0.950, *P* < 0.001), indicating that the thinner the choroid, the higher the risk of RDR. Similar results were obtained for each of the other ETDRS subregions, with multivariable-adjusted RRs ranging from 0.891 to 0.930, indicating that each 10-µm CT decrease was associated with a 7.0% to 10.9% increase in RDR risk (Supplementary Table S3).

### Associations Between CT Measurements and New-Onset DME

Univariable logistic regression analyses demonstrated that incident DME was related to age, duration of diabetes, HbA1c, SBP, TG, and DR status at baseline (Supplementary Table S4). Average CT was not significantly associated with risk of DME (RR = 0.998, 95% CI = 0.886–1.124, *P* = 0.972). After adjusting for other factors, the higher odds of DME were related to younger age (RR = 0.27, 95% CI = 0.09–0.75, *P* = 0.012) and presence of DR at baseline (RR = 65.62, 95% CI = 11.44–376.58, *P* < 0.001).

### Prognostic Value of Average CT in Predicting RDR

**Figure 3.**
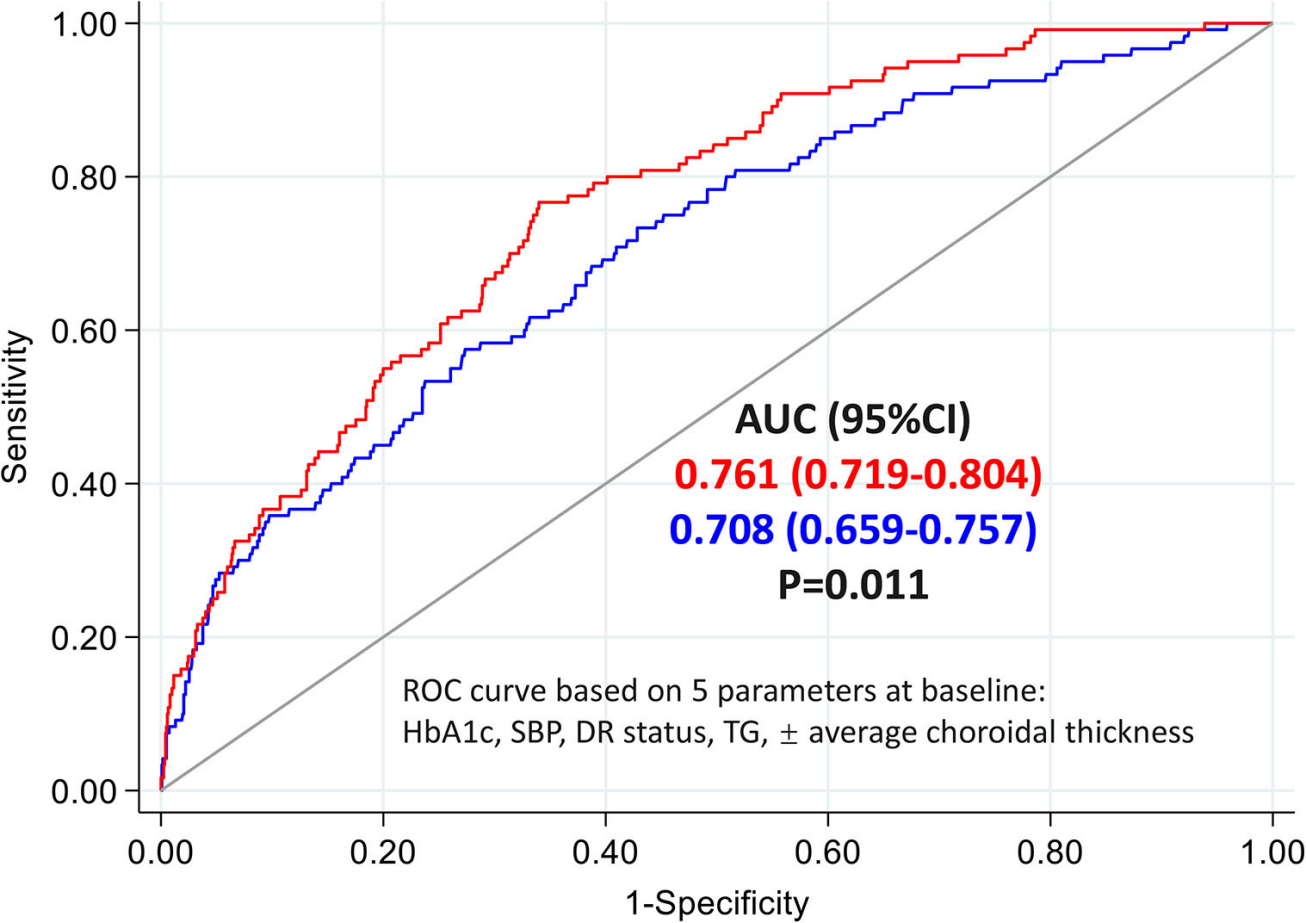
Receiver operating characteristic (ROC) curves of the prediction models for discriminating high-risk RDR people from stable people. The blue line indicates the conventional model including HbA1c, systolic blood pressure, triglycerides, and diabetic retinopathy status at baseline. The red line indicates the conventional factors plus average choroidal thickness. RDR = referable diabetic retinopathy.

Figure 3 shows the performance of the independent predictors (see Table 3) in discriminating the RDR high-risk subjects from non-RDR subjects before 2 years. The AUC of the ROC was 0.708 (95% CI = 0.659–0.757) for the conventional model and 0.761 (95% CI = 0.719–0.804) for a new model that added average CT to the conventional model, with significant difference (*P* = 0.011). The average CT also improved the NRI (0.464 ± 0.096, *P* < 0.001) and IDI (0.0321 ± 0.0068, *P* < 0.001) measures for 2-year RDR risk, indicating significant improvement in discriminatory power after adding CT to the established factors model.

## Discussion

As one of the most metabolically active and vascular-rich tissues in the body, the choroid has been associated with a variety of diseases, but no longitudinal study has been done to elucidate the relationship between CT and DR. Using state-of-the-art SS-OCT, this 2-year follow-up study with a large sample size found that CT was independently associated with RDR occurrence, and the thinner the CT, the higher the probability of RDR. CT enhanced the discriminative power for identifying people at high-risk for incident RDR beyond the conventional factors. To the best of our knowledge, this study provides the first longitudinal evidence of the predictive value of CT for DR onset and progression.

Only five previous studies have used SS-OCT and an automatic algorithm to evaluate average CT, and all of them had a cross-sectional design (Table 4).^6^^,^^13^^–^^16^ These studies arrived at mixed results, with some reporting reduced average CT in diabetes or DR and others not noting these associations. By using a random model, the pooled estimations of these studies demonstrated that the average CT was reduced by -14.5 µm in patients with NDR compared with healthy controls and by -7.6 µm in patients with any DR compared with patients with NDR. However, it is important to note that the cross-sectional designs prevented inferring an association. In addition, only two of these studies corrected for known confounding factors, such as HbA1c, AL, and diastolic blood pressure associated with DR. All these factors were significantly associated with CT and could have led to bias and inaccurate results. Using a longitudinal design with a relatively large sample size, the present study broadens these previous studies by demonstrating for the first time that the choroid is associated with DR progression.

**Table 4.** Previous Cross-Sectional Studies on Changes of Average Choroidal Thickness Based on SS-OCT and Automatic Algorithm

			Number of Eyes			Estimated Difference (95% CI)		
Author (Year)	Country	Device	Ctr	NDR	DR	NDR Versus Control	Any DR Versus NDR	Adjusted Factors
Kim (2018)	Korea	Triton	45	30	89	−47.8 (−83.5 to −12.1)	18.1 (−3.8 to 40.0)	None
Horváth (2018)	Hungary	Triton	46	17	–	−33.8 (−69.5 to 1.9)	–	None
Laíns (2018)	USA	Atlantis	50	27	133	23.3 (−7.3 to 53.9)	−47.9 (−63.5 to −32.3)	None
Huang (2020)	China	Atlantis	1027	946	304	−14.5 (−21.1 to −7.9)	−11.2 (−19.6 to −2.8)	Age, sex, AL, BMI, SBP, DBP, TC, TG, HbA1c, and FBG
Wang (2020)	China	Triton	–	1080	267	–	7.6 (−2.0 to 17.3)	Age, sex, AL, duration, BMI, SBP, DBP, TC, and HbA1c
**Inverse-variance pooled estimate**						−**14.5 (**−**20.7 to** −**8.3)**	−**7.6 (**−**13.3 to** −**1.9)**	

Ctr = control; NDR = non-diabetic retinopathy; AL = axial length; BMI = body mass index; SBP = systolic blood pressure; DBP = diastolic blood pressure; FBG = fasting blood glucose; TC = total cholesterol; TG = triglyceride.

CT indirectly reflects choroidal perfusion. In guinea pig eyes, CT was found to be highly correlated with choroidal perfusion values (*r* = 0.95, *P* < 0.001).^17^ In healthy subjects, CT was also found to be significantly correlated with choriocapillaris parameters, including choriocapillaris flow deficit (CC FD) density (*r* = 0.290, *P* = 0.021) and CC FD number (*r* = -0.312, *P* = 0.013).^18^ Using SS-OCT angiography, Zheng et al.^19^ reported that CT was stably correlated with choriocapillaris vessel density (*r* = 0.70–0.77) in patients with AMD with or without reticular pseudodrusen. In healthy subjects and patients with glaucoma, other studies have found that CT was independently associated with ocular perfusion pressure after adjusting for refractory errors, suggesting that CT may indirectly reflect ocular perfusion status.^20^^,^^21^ Ocular pulse amplitude (OPA), which provides information on intraocular blood flow, is considered to be an indicator of choroidal perfusion, and Demirok et al.^22^ reported that OPA was strongly correlated with CT in healthy subjects (*r* = 0.481, *P* = 0.001). Novais et al.^23^ used color Doppler flowmetry to measure retrobulbar blood flow, and they found a negative correlation between CT and retrobulbar arterial resistance index; these results imply that decreased choroidal blood flow could explain CT thinning in healthy subjects. The present study focused on the additive value of CT in discriminating subjects at high-risk for RDR from those with a lower risk. Future studies are needed to evaluate the impact of CC FD, OPA, and retrobulbar dynamics on RDR development.

In addition to CT measurements, the choroidal vascularity index (CVI) is considered to be a powerfully OCT-derived choroidal parameter. CT reflects the entire structure of the choroidal vascular system without differentiating between the stromal and luminal components. In addition to CT measurements, the CVI, which is the ratio of vascular area to total choroidal area, is considered to be a powerfully OCT-derived choroidal parameter.^24^ Gupta and colleagues found that both CT and CVI were significantly correlated with DR severity, and the lowest CT and CVI were in PDR eyes, suggesting that choroidal ischemia and hypoxia might induce retinal neovascularization.^25^ In a study examining the alterations of choroid in patients with DM, the CT and CVI in non-DR patients were significantly lower than in healthy controls, and this finding could indicate that choroidal changes precede retinopathy.^26^ By using SS-OCT and OCT angiography, we found that each 1-µm increase in average CT was significantly associated with decrease of CC FD% -0.004 (95% CI = -0.007 to -0.001, *P* = 0.012; Supplementary Fig. S1). These findings show that both CT and CVI derived from OCT might reflect choroidal changes that precede retinopathy.

The mechanism by which the choroid contributes to DR progression is unclear. The choroid provides oxygen and nutrients to the outer layer of the retina and retinal pigment epithelium (RPE). Hyperglycemia leads to choriocapillaris loss, increased vessel resistance, reduced choroidal perfusion, and retinal tissue hypoxia. Choroidal thinning indicates diabetes-related hemodynamic changes, such as blood flow disturbances and vascular endothelial dysfunction.^27^ Choroidal thinning for blood flow disturbances might lead to ischemia and hypoxia in the RPE and the subsequent production of vascular endothelial growth factor that leads to choroidal neovascularization.^28^^,^^29^ Lower CT may also be a sign of impaired self-regulation of small arteries, and this process could lead to capillary wall dilatation (microaneurysms), leakage (edema and hard exudate), and rupture (hemorrhage). In addition, choroidal ischemia and hypoxia lead to inflammation and oxidative stress, which can further drive DR progression and serve as the mechanism by which CT is associated with DR progression.^30^^,^^31^ Accordingly, choroidal thinning may be the primary cause of retinal ischemia, in which case CT would be significant in predicting the extent of DR lesions and providing new ideas for clarifying the specific mechanisms of DR progression.

The role of choroidal indicators in DME remains controversial. Kim et al.^16^ found no significant difference in CVI between patients with and without clinically significant macular edema. However, Gupta et al.^25^ reported a significant reduction in CVI in patients with diabetic macular edema. In this study, we did not observe a significant association between CT and incident DME. Only 7 (0.52%) patients developed DME during the 2-year follow-up, which is consistent with the incidence listed in previous reports. It has been estimated that 0.55% and 6.5% of people with type 2 diabetes developed DME within 2 years, according to the US Multi-Ethnic Study of Atherosclerosis and the Indian Sankara Nethralaya-Diabetic Retinopathy Epidemiology and Molecular Genetics Study, respectively.^32^^,^^33^ Future studies with larger samples are needed to confirm whether CT or CVI is a biomarker for DME.

This study has several important advantages. First, because we collected seven-field fundus color photographs to assess DR both at baseline and during follow-up, less potential existed for missing or misclassifying mild disease. Second, the prospective, longitudinal study design included a large sample of patients with DM, giving us sufficient statistical power to analyze the association between CT and RDR occurrence. Third, this study used SS-OCT for choroidal quantification. Whereas traditional SD-OCT is limited by its low choroidal resolution and errors from manual measurements, SS-OCT provides a clearer boundary of the choroid–scleral interface by using a longer laser wavelength (1050 nm) that reduces the dispersion caused by the RPE. In addition, the high imaging speed and automated measurements greatly improve accuracy, reducing manual errors and providing more reliable results. Fourth, to remove the confounding effects of intraocular VEGF injections and retinal laser photocoagulation on CT, we enrolled patients with diabetes with no history of ocular treatment. We also adjusted for other confounding factors, including serum creatine, which has been widely neglected in previous studies.

This study has some limitations. First, the subjects were Chinese patients with type 2 DM. It has been shown that CT measurements may differ by ethnicity and type of diabetes, so the conclusions should not be generalized to other races or to type 1 DM. Second, only 2 years of follow-up does not allow for knowing whether CT has a longer-term effect on DR. Third, in choroidal studies, circadian rhythm should be considered. Usui and colleagues reported that CT varied between 270 µm at 3 PM and 290 µm at 3 AM, with a circadian variation of approximately 7%.^34^ Similarly, Tan et al.^35^ reported a circadian variation in CT between 372 µm and 341 µm, which is approximately 8%, and Burfield et al.^36^ reported a circadian variation in CT of 5.8 ± 13.44 µm, with a magnitude of approximately 7.7%. In this study, the OCT imaging was taken at various times of the day. Because these examinations were performed randomly, a mild measurement bias from circadian variations might exist. Furthermore, the multivariable model that accounted for OCT examination timing obtained results consistent with the primary analyses (see Supplementary Table S3). Fourth, a clear choroid–scleral junction is a prerequisite for accurate CT measurements. Several CT posterior boundaries have been proposed, and the visibility of the choroid–scleral interface (CSI) has a significant impact on CT measurements. Automatic choroidal outer border segmentation has been reported to favor the identification of the posterior pole of the choroidal vessels by SS-OCT. In this study, two investigators reviewed all OCT scans, and the CSI required manual correction in some cases, which might have introduced systematic errors, albeit likely very small. Although we excluded patients with moderate to severe cataracts, individual differences in lens density could have also affected CT measurements. Although the faster scanning speed of SS-OCT can mitigate motion artifacts to some extent, the technology is still sensitive to axial motion artifacts.^37^ Finally, no defined threshold for CT exists because CT overlaps with “disease” and “absence of disease,” making it impossible to identify individuals at risk. Assessing CT changes over time at the individual level is a better way to identify individuals at risk, and we are currently conducting research in this area to evaluate this approach.

## Conclusion

This study is the first longitudinal study of diabetes based on SS-OCT in a large sample to find that CT thinning is independently associated with RDR occurrence and is an essential imaging marker for DR progression. Automated quantitative assessment of CT using SS-OCT can provide prognostic information about RDR risk in patients with type 2 DM. However, this finding requires further animal and molecular studies to examine the specific signal pathways and to further validate the results in populations with different ethnicities and diabetes types.

## Supplementary Material

Supplement 1
