# Supplementary material for: Macular Choroidal Thickness and the Risk of Referable Diabetic Retinopathy in Type 2 Diabetes: A 2-Year Longitudinal Study
Source: Invest Ophthalmol Vis Sci. 2022 Apr 14;63(4):9. doi: 10.1167/iovs.63.4.9 (PMC9034727; doi:10.1167/iovs.63.4.9)
Supplement: Supplement 1 [file iovs-63-4-9_s001.pdf]

Supplementary Figure 1. The relationship between average choroidal thickness (CT) and choriocapillaris flow deficit (CC FD%) in 3×3mm region (n=74).

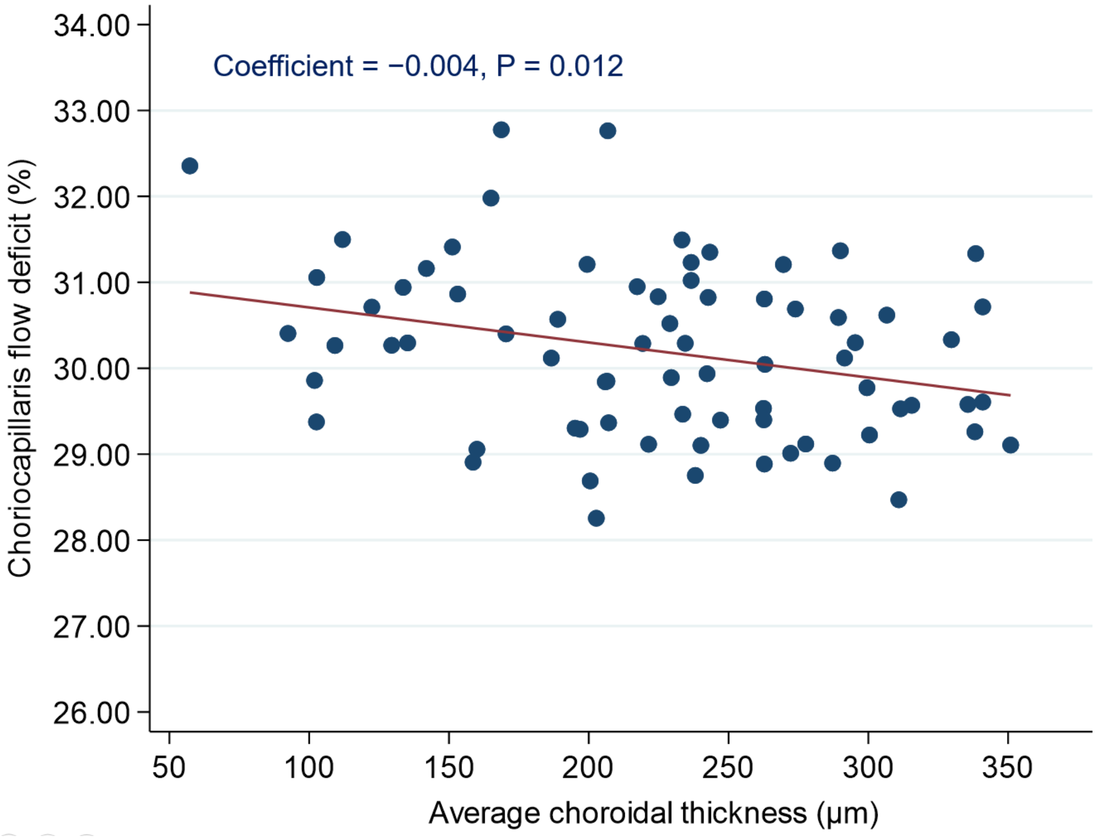

Supplementary Table 1. Baseline characteristics of the included and excluded subjects.

Characteristics	All	Include	Exclude	P-value
No. of subjects	1572	1345(85.56%)	227(14.44%)	-
Female, %	920(58.52%)	792(58.88%)	128(56.39%)	0.480
Age, year	64.2±7.6	63.8±7.4	66.2±8.3	<0.001
DM duration, year	8.5±6.6	8.4±6.5	8.6±7.0	0.689
HbA1c, %	6.73±1.21	6.74±1.17	6.66±1.40	0.365
SBP, mm Hg	134.1±17.9	133.6±17.8	137.0±18.4	0.009
DBP, mm Hg	71.1±10.6	70.8±9.9	72.9±13.9	0.006
Cholesterol, mmol/L	4.78±1.06	4.79±1.06	4.70±1.05	0.209
Creatinine, µmol/L	70.0±19.3	69.4±19.0	73.3±21.0	0.005
HDL-c, mmol/L	1.33±0.42	1.33±0.41	1.34±0.45	0.706
LDL-c, mmol/L	3.03±0.94	3.05±0.94	2.95±0.94	0.145
TG, mmol/L	2.30±1.60	2.29±1.63	2.33±1.38	0.759
CRP, mg/L	2.49±5.94	2.45±6.18	2.74±4.29	0.500
MAU, mg/mL	4.12±15.07	4.06±15.73	4.46±10.47	0.715
BCVA, logMAR	0.21±0.15	0.19±0.12	0.30±0.28	<0.001
IOP, mmHg	16.3±2.8	16.3±2.8	16.3±2.6	0.890
CCT, µm	547.0±31.2	546.8±31.2	548.1±31.0	0.563
Axial length, mm	23.6±1.2	23.4±0.9	24.7±1.9	<0.001
ACD, mm	2.5±0.5	2.5±0.5	2.7±0.7	<0.001
Len thickness, mm	4.7±0.3	4.7±0.3	4.7±0.4	0.020
Corneal diameter, mm	11.6±0.4	11.6±0.4	11.7±0.4	0.120
Any DR at baseline, %	77(4.90%)	65(4.83%)	12(5.29%)	0.770

DM=diabetes mellitus; SBP=systolic blood pressure; D=diopters; HDL-c=high-density lipoprotein cholesterol; CCT=central corneal thickness; DR=diabetic retinopathy.

Supplementary Table 2. Baseline distribution of choroidal thickness in macular region by incident of DME at 2-year follow-up.

Characteristics	All	Incident DME		
		No	Yes	P-value
Outer Superior, µm	204.3±65.0	204.4±65.0	191.7±69.7	0.607
Inner Superior, µm	214.4±68.8	214.4±68.9	202.7±56.2	0.654
Outer Temporal, µm	184.6±58.7	184.6±58.8	184.9±35.3	0.990
Inner Temporal, µm	206.9±65.5	206.9±65.6	203.7±46.6	0.897
Central field, µm	215.1±72.3	215.2±72.4	205.6±65.4	0.727
Inner Nasal, µm	202.0±74.2	202.1±74.2	196.1±68.8	0.833
Outer Nasal, µm	160.0±70.7	160.0±70.6	157.1±96.0	0.914
Inner Inferior, µm	201.7±74.4	201.6±74.3	214.3±90.0	0.653
Outer Inferior, µm	178.9±70.5	178.8±70.5	203.7±74.1	0.351
Average, µm	196.4±62.6	196.4±62.6	195.6±61.3	0.972

DME=diabetic macular edema.

Supplementary Table 3. Baseline choroidal thickness in ETDRS grids and 2-year risk of incident RDR.

Per 10-μm increase	Model 1*		Model 2†		Model 3‡	
	RR (95%CI)	P-value	RR (95%CI)	P-value	RR (95%CI)	P-value
Outer Superior	0.913(0.884 to 0.943)	<0.0001	0.904(0.874 to 0.936)	<0.0001	0.905(0.875 to 0.936)	<0.0001
Inner Superior	0.928(0.901 to 0.955)	<0.0001	0.916(0.888 to 0.946)	<0.0001	0.917(0.888 to 0.946)	<0.0001
Outer Temporal	0.900(0.867 to 0.933)	<0.0001	0.891(0.857 to 0.926)	<0.0001	0.891(0.857 to 0.926)	<0.0001
Inner Temporal	0.913(0.884 to 0.943)	<0.0001	0.905(0.875 to 0.937)	<0.0001	0.905(0.875 to 0.937)	<0.0001
Central field	0.930(0.904 to 0.957)	<0.0001	0.923(0.895 to 0.951)	<0.0001	0.922(0.895 to 0.950)	<0.0001
Inner Nasal	0.938(0.912 to 0.964)	<0.0001	0.931(0.905 to 0.959)	<0.0001	0.930(0.904 to 0.958)	<0.0001
Outer Nasal	0.934(0.906 to 0.963)	<0.0001	0.928(0.900 to 0.958)	<0.0001	0.927(0.898 to 0.957)	<0.0001
Inner Inferior	0.937(0.911 to 0.963)	<0.0001	0.931(0.904 to 0.959)	<0.0001	0.930(0.903 to 0.957)	<0.0001
Outer Inferior	0.930(0.902 to 0.958)	<0.0001	0.925(0.896 to 0.955)	<0.0001	0.923(0.894 to 0.954)	<0.0001

RDR=referable diabetic retinopathy.

*Univariable model

†Multivariable model adjusted for HbA1c, duration of diabetes, SBP, triglyceride, baseline status of retinopathy.

†Multivariable model adjusted for HbA1c, duration of diabetes, SBP, triglyceride, baseline status of retinopathy, and OCT imaging time of the day.

Supplementary Table 4. Univariable and stepwise multivariable logistic analyses of the potential predictors for the incident DME.

Parameters at baseline	Univariable model		Stepwise multivariable model	
	RR (95%CI)	P-value	RR (95%CI)	P-value
Per 10-year increase in age	0.36 (0.16 to 0.82)	0.015	0.27 (0.09 to 0.75)	0.012
Male vs female	1.08 (0.24 to 4.82)	0.925		
Per 1-year increase in diabetes duration	1.08 (0.99 to 1.18)	0.096		
Per % increase in HbA1c level	1.58 (1.09 to 2.29)	0.016		
Per 1-mmHg increase in systolic blood pressure	0.96 (0.92 to 1.00)	0.041		
Per 1-mmHg increase in diastolic blood pressure	0.94 (0.87 to 1.02)	0.146		
Per 1-mmol/L increase in total cholesterol	1.04 (0.52 to 2.08)	0.912		
Per 1-mmol/L increase in serum creatinine	0.98 (0.94 to 1.03)	0.379		
Per 1-mmol/L increase in HDL-c level	1.03 (0.17 to 6.18)	0.978		
Per 1-mmol/L increase in LDL-c level	1.29 (0.61 to 2.76)	0.508		
Per 1-mmol/L increase in TG level	0.34 (0.10 to 1.11)	0.074		
Per 1-mg/L increase in C-reactive protein	0.80 (0.45 to 1.41)	0.435		
Per 1-mg/mL increase in microalbuminuria	0.89 (0.60 to 1.32)	0.560		
Per 1-mmHg increase in intraocular pressure	1.21 (0.94 to 1.55)	0.140		
Per 1-μm increase in central corneal thickness	1.02 (1.00 to 1.04)	0.092		
Per 1-mm increase in axial length	1.01 (0.44 to 2.32)	0.978		
Per 1-mm increase in anterior chamber depth	0.78 (0.13 to 4.86)	0.792		
Per 1-mm increase in lens thickness	0.91 (0.09 to 8.97)	0.932		
Per 1-mm increase in corneal diameter	0.47 (0.10 to 2.27)	0.347		
DR status at baseline (Present vs Without)	53.25 (10.12 to 280.10)	<0.001	65.62 (11.44 to 376.58)	<0.001
Per 10-μm increase in average choroidal thickness	0.998 (0.886 to 1.124)	0.972		

DME=diabetic macular edema.
